# Desperation and inequality increase stealing: evidence from experimental microsocieties

**DOI:** 10.1098/rsos.221385

**Published:** 2023-07-19

**Authors:** Setayesh Radkani, Eleanor Holton, Benoît de Courson, Rebecca Saxe, Daniel Nettle

**Affiliations:** ^1^ Department of Brain and Cognitive Sciences, Massachusetts Institute of Technology, Cambridge, MA 02139, USA; ^2^ McGovern Institute for Brain Research, Massachusetts Institute of Technology, Cambridge, MA 02139, USA; ^3^ Newcastle University Population Health Sciences Institute, Newcastle University, Newcastle upon Tyne, UK; ^4^ Max Planck Institute for the Study of Crime, Security and Law, Freiburg, Germany; ^5^ Institut Jean Nicod, Département d’études cognitives, École Normale Supérieure, Université PSL, EHESS, CNRS, Paris, France

**Keywords:** cooperation, inequality, crime, punishment, desperation, economic games

## Abstract

People facing material deprivation are more likely to turn to acquisitive crime. It is not clear why it makes sense for them to do so, given that apprehension and punishment may make their situation even worse. Recent theory suggests that people should be more willing to steal if they are on the wrong side of a ‘desperation threshold’; that is, a level of resources critical to wellbeing. Below such a threshold, people should pursue any risky behaviour that offers the possibility of a short route back above, and should be insensitive to the severity of possible punishments, since they have little left to lose. We developed a multi-round, multi-player economic game with a desperation threshold and the possibility of theft as well as cooperation. Across four experiments with 1000 UK and US adults, we showed that falling short of a desperation threshold increased stealing from other players, even when the payoff from stealing was negative on average. Within the microsocieties created in the game, the presence of more players with below-threshold resources produced low trust, driven by the experience of being stolen from. Contrary to predictions, our participants appeared to be somewhat sensitive to the severity of punishment for being caught trying to steal. Our results show, in an experimental microcosm, that some members of society falling short of a threshold of material desperation can have powerful social consequences.

## 1. Introduction

People with few material resources are more likely to commit acquisitive crimes; that is, crimes that lead to material gain for the perpetrator [1,2]. Two important questions about this association are, first, whether it is causal; and, second, why it exists. The present study addresses both of these questions.

On the first question, it seems logical that lack of resources would cause people to try to acquire them, including through illegitimate means. However, the observed associations could always be explained in non-causal ways. For example, both the lack of material resources and a propensity to illegitimate activity could be explained by some third individual or structural variable. Researchers have tried to identify causality by studying the impact on crime of putatively exogenous shocks to people's resource levels, such as fluctuations in unemployment or wage rates at the bottom of the income distribution [1–3]. Nonetheless, in observational data, causal inference always remains tentative.

It is neither feasible nor ethical to manipulate people's actual material situation for the sake of research. In such situations, social scientists sometimes turn to ‘experimental microsocieties' to test causal hypotheses [4–6]. In such designs, volunteer participants interact in experimental social situations carefully designed to capture key features of the real-world situation of interest. Hypothesized causal factors can be manipulated, and behavioural outcomes measured. Showing that a factor has the hypothesized effect within the experimental microcosm by no means guarantees that the factor is a causal driver in real societies, but it is a useful proof of principle that it could be.

The second question is why people short of resources would be more likely to try to acquire them illegitimately. Why not rectify a lack of resources through legitimate means? Classic economic theories assumed that the returns to legitimate economic activity tend to be worse for people with few resources, making illegitimate activity relatively more attractive for them [7]. However, acquisitive crimes often generate very small amounts of resource (see [8]). Given a non-zero probability of apprehension, and the huge cost that would entail, the expected return may well be negative. People committing acquisitive crimes may therefore be making themselves even worse off in the long run, at least in expectation. However, while acquisitive crime might be a worse option than legitimate economic activity on average, it also has a bigger variance in possible payoffs. While the worst-case outcome (apprehension) is very negative, the best-case outcome is an immediate positive impact on resources. Thus, one hypothesis is that, under low resources, the possible immediate upside becomes more attractive, and/or people become less sensitive to the possible downside. Qualitative studies suggest that acquisitive crime is indeed very often proximally motivated by the perceived pressing need for ‘fast cash’ [9].

A scenario that would make the possibility of ‘fast cash’ attractive is the existence of a ‘desperation threshold’. A desperation threshold is a critical level of resources such that having any less, even transitorily, is gravely and permanently damaging. In foraging theory, for example, the point of starvation is a desperation threshold: an animal close to this point should take any action that might yield sufficient immediate calories; if it works, they escape starvation, and if it does not, they cannot be any worse off than starving [10]. For humans, a desperation threshold need not be actual starvation. It could be, for example, the inability to pay rent, leading to the loss of a place to live. We recently created a theoretical model in which agents with variable resources choose to cooperate with others, steal from others, or avoid interaction [11] (we henceforth refer to this paper as CN). CN stipulated that, because of the possibility of apprehension, stealing has a negative expected return, but a non-zero probability of producing substantial immediate resources. Under these assumptions, the model showed that stealing is the favoured option only when: (a) there is a desperation threshold level of resources in the agent's utility function; and (b) the agent's current resources are at or below this threshold. When above the threshold, agents should either cooperate or avoid interaction, depending on the prevalence of stealing in the surrounding population. Further, below-threshold stealing should be insensitive to the magnitude of the penalty for getting caught, since below-threshold agents are in such dire straits that a larger punishment cannot make things much worse.

As well as deriving optimal strategies for individual decision makers, the CN model provided a bridge to understanding aggregate-level phenomena such as the relationship between economic inequality, crime and trust. CN simulated populations of agents following the optimal strategies predicted by their model. Populations with more unequal distributions of resources developed higher levels of crime, as long as there was a desperation threshold, and the increased inequality meant a greater fraction of agents had below-threshold levels of resources. As a consequence, in unequal populations, cooperation became rare even among agents with above-threshold resources.

The CN model provides an account of how people are expected to behave in the presence of a desperation threshold. However, theory alone cannot demonstrate that humans are indeed psychologically responsive to such thresholds in the predicted way. Observational evidence on patterns of crime is consistent with CN's aggregate-level predictions. For example, greater inequality in the distribution of resources is associated with higher crime rates [12] and lower trust [13]; and researchers have generally concluded that crime decisions are insensitive to severity of punishment [14]. However, as already discussed, experimental research would permit a stronger test of the model's causal structure. There is a limited amount of prior relevant experimental work. Mishra & Lalumière [15] studied a range of tasks involving choice between a risky and a safe option, in the presence of a ‘need’, a minimum yield that the participant had to achieve or else fail the task. As the probability of meeting the need through the safe option reduced, participants shifted to riskier options (see also [16]). The tasks used in those experiments were non-social. The risky options had a greater variance in possible payoff, but the participant's choice of action had no consequences for other people's outcomes. In our paradigm, the risky choice is explicitly framed as stealing, and involves a chance of benefiting at by taking resources from another participant, and a chance of being caught and punished.

In the present project, we created an experimental microsociety paradigm within which we could study the impact of resource shortfall and inequality on the decision to steal from others. Hence, we could test both the individual-level and aggregate-level predictions arising from the CN model. Our paradigm consists of an incentivized multi-round, multi-player economic game. Participants play for ‘energy points’, which are converted to money at the end of the game. In the default condition, the final payment is subject to a penalty for every round the participant had a points level below a given threshold (the desperation threshold). This penalty is sufficiently large that even a few rounds with below-threshold points will reduce game earnings to zero. Mirroring the situation modelled in CN, participants have three behavioural options in each round: working alone, which has no energy points yield, but no risk; cooperating, which yields a small gain as long as other players also cooperate; and stealing, which may yield a larger gain, or may lead to getting caught and suffering a large loss. In studies 1 and 2, we test the basic predictions that individuals should be more likely to steal when their energy points level falls below the desperation threshold, and that, when above threshold, the choice between cooperating and working alone should depend on the prevalence of stealing by others in the population. In study 3, as well as replicating the results of studies 1 and 2, we test the further prediction that below-threshold stealing will be insensitive to the magnitude of punishment. Finally, in study 4, we test the population-level prediction that greater inequality in the allocation of energy points will produce a greater prevalence of stealing, because it places some individuals into structural desperation.

## 2. Methods overview

In this section, we describe our general methods. Methods sections for individual studies detail only departures from the general protocol.

### 2.1. Participants

Participants were adult volunteers (UK resident in studies 1 and 2; US resident in studies 3 and 4) recruited via research participation platform Prolific. Prolific members sign up to be notified of research studies, which they complete remotely for small payments. Use of platforms like Prolific is widespread in experimental psychology. They can rapidly produce large datasets. These have been shown in controlled comparisons to be of high quality and to replicate the results of in-person studies in a number of cases [17–19]. Of US and UK Prolific members active in last 90 days (6 April 2023), 57.7% had declared at registration that they had university degrees, against 42.3% who did not; and 56.3% placed themselves on rungs 6–10 of the Macarthur ladder of subjective socioeconomic status [20], against 43.6% on rungs 1–5. Thus, the Prolific pool is more diverse than typical student participant pools, but over-represents university educated and higher socioeconomic status groups relative to the general population (e.g. university degrees among 18–64 year olds: UK, 41.3%; USA, 39.5% [21]). Age, gender and employment statuses of our samples for each study are reported in table 1.

**Table 1.** Pre-registration and sample information. Numbers for demographic categories do not sum to *N*_included_ due to individuals who did not provide demographic information to Prolific.

study	pre-registration	*N*_recruited_	*N*_included_	demographics
1	https://osf.io/8zbp6	96	84	53 women, 27 men
				43 in work, 23 students
				mean age 30.15 (s.d. 8.78)
2	https://osf.io/snd54	180	160	81 women, 78 men
				84 in work, 35 students
				mean age 33.67 (s.d. 11.43)
3	https://osf.io/ybvea	258	183	83 women, 97 men
				113 in work, 47 students
				mean age 33.37 (s.d. 10.34)
4	https://osf.io/a86mh	648	573	298 women, 267 men
				263 in work, 128 students
				Mean age 35.43 (s.d. 12.91)

### 2.2. Experimental game

**Figure 1.**
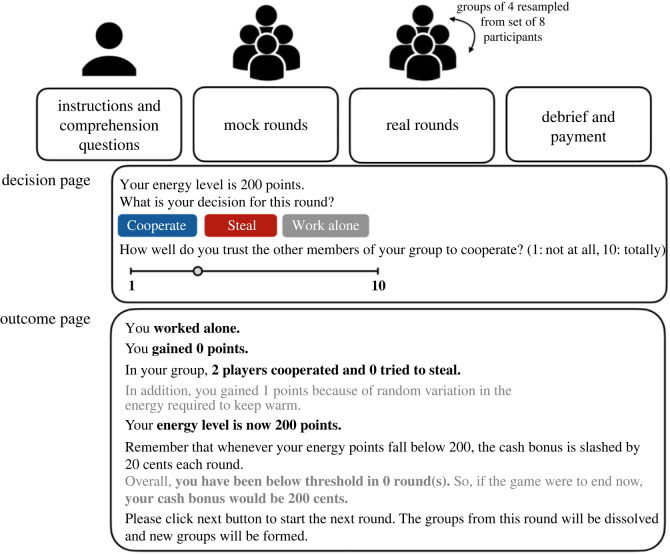
Design of the experimental game. All four studies followed the same basic procedure. After joining the experiment, each participant read the instructions and answered comprehension questions. The game started with five (studies 1 and 2) or four (studies 3 and 4) mock rounds which did not affect the player's cash bonus. These were followed by 8–16 real rounds (uniform distribution, studies 1–3) or 12 rounds (study 4). In each round, two groups of four participants were resampled from the set of eight participants. Each participant indicated their level of trust and decided to ‘cooperate’, ‘steal’ or ‘work alone’. After all players had made their decision, they saw an outcome page showing the aggregate decisions of the other players, including whether stealing attempts were successful or not. Text written in grey indicates information that was different between studies 1–2 and 3–4.

All four studies used variants of the same experimental game (for overview, see figure 1; differences between studies are summarized in table 2). The game was coded in oTree [22] and mounted on an Internet server. Participants took part in sets of eight. In each round, interaction groups of four players were drawn up at random from the set. Each player was asked to choose an action from three alternatives with different energy point consequences: ‘*cooperate’*, which produced a small gain as long as at least one other player cooperated and no-one stole, but could produce a small loss if another player stole successfully; ‘*steal*’, which, if at least one other player cooperated, would with equal probability produce either a large gain (stealing successfully), or a very large loss (getting caught); or ‘*work alone*’, which neither gained nor lost points. Besides choosing an action, players were asked how much they trusted the other players to cooperate (on a 10-point scale).

**Table 2.** Points values and cash conversion rates used in each of the four studies.

study	cooperate payoff	steal payoff	work alone payoff	desperation threshold^a^	starting points levels	points to cash conversion rate	cash penalty^b^
1	successful: +5	successful: +20	0	100	100	2 pence/point	−50 pence/round
2	stolen	punished: −40					
3	from: −5	successful: +10		200	[180, 190, 200]	1 cent/point	−20 cents/round
		punished: [lenient: −15; harsh: −30]					
4		successful: +10			equal: 205		
		punished: −15			unequal: [180, 190, 220, 230]		

^a^Studies 2–4 included a no-threshold condition in which this was not applied.

^b^For every round in which the participant's points were below threshold level in the threshold condition.

After each round, the players were directed to an outcome page showing the decisions of other players as well as their own (figure 1). Participants always knew their own energy points level, but no information was ever given about the energy points levels of other players. In studies 1 and 2, after each round, an additional random perturbation was applied to each player's energy points level (reported to the participant in the outcome page), drawn from a discretized Gaussian distribution with mean 0 and standard deviation 3. Participants were not aware exactly how many rounds there would be.

After the final round, energy points were converted to pounds or dollars (for rates of exchange, see table 2). In the desperation threshold conditions, a penalty was applied to the converted sum, prior to payment, for every round the participant had had an energy points level below a threshold level (100 points in studies 1 and 2; 200 points in studies 3 and 4). This penalty rule was explained prior to the game. Participant earnings from the game were between £0 and £3.24 (study 1); £0 and £4.10 (study 2); $0 and $2.90 (study 3); $0 and $2.95 (study 4). In addition, they were paid a ‘show up’ fee of £2 (studies 1 and 2), $4 (study 3) or $4.20 (study 4). The number of rounds played by each set of 8 players was variable; but the sessions typically lasted around 25 min. Any player who became inactive was automatically set to make ‘work alone’ decisions. Players who completed fewer than 4 real rounds were excluded from analysis. Four rounds constitute half a session for the shortest sessions.

### 2.3. Data analysis

Data and analysis code are available at: https://osf.io/kf87e/. Data were analysed using Bayesian generalized linear mixed models using the brms R package [23]. Priors on parameters were N(0, 1). We report parameter estimates, their 95% credible intervals (CI), and Bayes factors (BFs) for each parameter (Savage–Dickey density ratios). Pre-registered predictions are identified with the letter P. We interpret strength of evidence from BFs using the categories given by Andraszewicz *et al*. [24]. We also repeated all analyses using frequentist generalized linear mixed models, which produced very similar conclusions. Depending on the prediction, the unit of analysis was either the individual decision, or the set (i.e. counting behaviours and averaging trust across the eight players). Unless otherwise stated, all analyses control for round. Rate of stealing and number of stealers refer to attempts rather than successful attempts.

## 3. Study 1

Study 1 tested the most basic behavioural predictions arising from CN. These were that, in a game with a desperation threshold, individuals should steal more when their resource levels are below than above it; and that exposure to stealing will lead individuals to avoid interaction rather than cooperating. Since trust is a regulatory psychological variable tracking the propensity of others to be good co-operators [25], we predicted that, in the microsocieties created in the experiment, more individuals in desperation should produce more stealing, and therefore less trust and less cooperation. In study 1, all players began the game with 100 points, exactly equal to the threshold. To produce variation in resources, we relied on small random shocks to participants' resource levels, which we applied each round, as well as the consequence of decisions taken in earlier rounds.

### 3.1. Results

Tests of pre-registered predictions are reported in table 3. The odds of stealing were more than twice as high when individuals had a points level below the threshold than above (table 3, P1.1; figure 1*a*). At the set level, average trust was lower where there had been more below-threshold players in the previous round (table 3, P1.2; figure 1*b*). This association was partially mediated by stealing: when there were more players below the threshold, there were more stealers (*β* = 0.49 s.d. change for 1 s.d. increase in number of players below, 95% CI 0.20 to 0.74, BF 45.70, very strong support); and when there were more stealers, trust was lower on the next round (*β* = −0.20, 95% CI −0.32 to −0.08, BF 18.03, strong support). The mediation pathway (*β* = −0.08, 95% CI −0.17 to −0.02) accounted for 23% of the total effect of players below threshold on subsequent trust. When not stealing, players were more likely to cooperate rather than working alone if their trust was higher (table 3, P1.3; figure 1*c*).

**Table 3.** Pre-registered predictions and corresponding results, study 1.

number	prediction	result
P1.1	individuals will be more likely to steal if their points level is below threshold than above	strong supporting evidence, OR 2.63 (95% CI 1.37 to 4.96), BF 22.64
P1.2	trust will be lower in sets containing more individuals below the threshold, due to a higher rate of stealing	extreme supporting evidence for trust, *β* = −0.29 s.d. change in trust for 1 s.d. increase in number below threshold (95% CI −0.44 to −0.15), BF 544.23. Partial mediation by stealing (see text)
P1.3	trust will predict choosing to cooperate over working alone: high trust will make the choice of cooperation more likely	extreme supporting evidence, OR for 1 s.d. increase in trust 3.56 (95% CI 2.44 to 5.32), BF > 1000

### 3.2. Discussion

Study 1 confirmed our central prediction: people whose points level was below the threshold were more likely to steal. Stealing in turn reduced trust within the set, and lower trust prompted people who were not stealing to switch from cooperation to working alone. The mediation of the association between number of people below the threshold and trust by the number of people stealing was only partial. However, this makes sense. First, the number of stealers is a property of the set of eight players: individual players may have experienced more or less stealing depending on which interaction groups they had happened to be in. Second, the analysis only used stealing in the previous round, whereas players may have been integrating their experience across multiple rounds. Finally, even in the absence of stealing, the decisions of other players to work alone rather than cooperate provides information about trust. Thus, our number of stealers variable only partially captured the information available to individual participants about the trust and trustworthiness of other players.

Although our three pre-registered predictions were all strongly supported, there are design issues limiting inferences about the causal impact of the threshold on stealing. First, given that all players began the game on 100 points, being below the threshold was completely confounded with having lost points relative to the starting position. Losses relative to a reference point are associated with risk-seeking [26]. This generalization is itself interesting in relation to the claims of CN, if reference points tend to be interpreted as representing a minimum acceptable level of resources. However, study 1 has not demonstrated that being below the threshold as defined within the game, as opposed to merely having lost relative to the starting point, increases stealing. Second, having an energy level below the threshold is partly endogenous: players could end up there as a consequence of (unsuccessfully) stealing, or of being (successfully) stolen from. Thus, some of the excess stealing below 100 points might represent a consistent propensity to steal, or a retaliatory response to being stolen from, rather than a causal consequence of the presence of a desperation threshold. Study 2 therefore aimed to address these inferential limitations.

## 4. Study 2

Study 2 sought to replicate study 1, but with the addition of a no-threshold condition. In this condition, all game payoffs and actions were the same, but there was no special financial penalty for having a points level below 100. Therefore, any excess stealing below 100 points (relative to above 100 points) in the *no-threshold* condition reflects the response to loss, retaliation, and/or a general propensity to steal. If players with fewer than 100 points increase their stealing more dramatically in the threshold condition than in the no-threshold condition, we can make the inference that the threshold itself has a causal impact on stealing.

### 4.1. Methods

#### 4.1.1. Experimental game

The game was as for study 1, but half the sets, and therefore half the participants, were run in a no-threshold condition, where final points were converted to cash at £0.02 per point with no penalty for rounds below 100 points, and no mention of a threshold in the instructions.

### 4.2. Results

**Figure 2.**
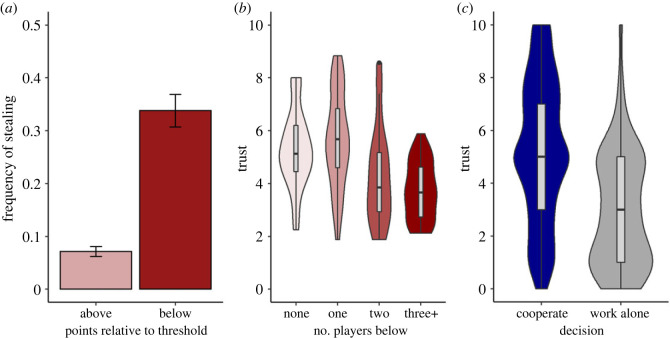
Results of study 1. (*a*) Frequency of stealing, by whether player's energy points level was currently above or below the threshold of 100. Error bars represent one standard error of the proportion. (*b*) Trust by the numbers of players below threshold in the previous round. Boxes show median and inter-quartile range of the mean for each set of eight players, and violins show the density of the data. (*c*) Distribution of trust ratings prior to choosing to cooperate, or to work alone.

**Figure 3.**
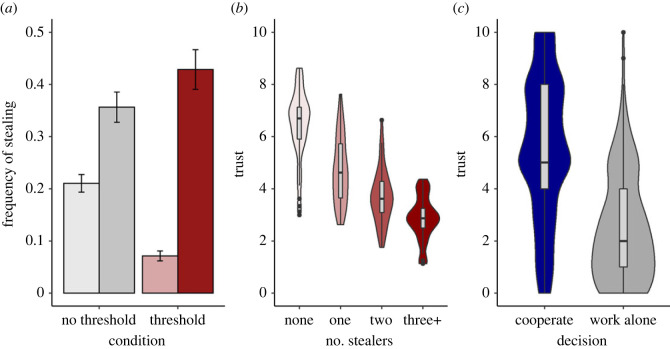
Results of study 2. (*a*) Frequency of stealing, by whether player's energy points level was currently above 100 (light) or below 100 (dark), and condition. Error bars represent one standard error of the proportion. (*b*) Trust (mean of the eight players' ratings) by the number of players stealing in the previous round, threshold and no-threshold conditions combined. Boxes show median and inter-quartile range of the set mean, and violins show the density of the data. (*c*) Distribution of trust ratings prior to choosing to cooperate, or to work alone.

Regardless of condition, people were more likely to steal when they had fewer than 100 points (figure 2*a*). However, there was also anecdotal evidence of an interaction between condition and having fewer than 100 points (P2.1 table 4; figure 3*a*). Being below (compared to above) 100 points increased stealing more in the threshold condition (OR 3.77, 95% CI 2.04 to 6.94, BF > 1000, extreme evidence) than the no threshold condition (OR 1.72, 95% CI 1.02 to 2.89, BF 2.09, anecdotal evidence). However, this difference was driven at least as much by a lower rate of stealing when above 100 points in the threshold as compared to the no threshold condition (OR 0.37, 95% CI 0.11 to 1.38, BF 2.16, anecdotal evidence), as any increase in stealing when below 100 points (OR 1.25, 95% CI 0.58 to 2.60, BF 0.45, anecdotal evidence for the null).

**Table 4.** Pre-registered predictions and corresponding results, study 2.

number	prediction	result
P2.1	there will be an interaction between condition (threshold, no threshold) and points level (above, below 100) in predicting stealing	anecdotal supporting evidence. OR 1.94 (95% CI 0.92 to 4.07), BF 1.77 (though see text)
P2.2	trust will be lower where the rate of stealing is higher	extreme supporting evidence. *β* = −0.28 s.d. change in trust for 1 s.d. increase in number of stealers (95% CI −0.38 to −0.18), BF > 1000
P2.3	trust will predict choosing to cooperate over working alone: high trust will make the choice of cooperation more likely	extreme supporting evidence. OR for 1 s.d. increase in trust 6.87 (95% CI 4.73 to 10.23), BF > 1000

At the set level, average trust was lower where there were more stealers in the previous round (table 4, P2.2; figure 3*b*). Hence there was an indirect association between the number of players below 100 points in the previous round and trust (*β* = −0.16 s.d. change for 1 s.d. increase in number of players below, 95% CI −0.26 to −0.06, BF 8.82, moderate evidence). As in study 1, among those not stealing, higher trust predicted choosing to cooperate over working alone (table 4, P2.3; figure 3*c*).

### 4.3. Discussion

In study 2, we added a condition with no desperation threshold. We expected that having an energy level below, as compared to above, 100 points would be associated with more stealing, due to having lost relative to the starting point, retaliation, and consistent propensities to steal. There was indeed some evidence of this. Critically, we predicted that the effect of being below 100 points would be stronger than this when there was a desperation threshold. This was indeed the case, although the strength of evidence for the interaction was only anecdotal. Moreover, the nature of the interaction was not quite as predicted: the presence of a desperation threshold appeared to suppress stealing when above the threshold at least as much as it exacerbated stealing when below. Other findings from study 2 directly replicated study 1: exposure to stealing reduced trust, and lower trust increased the chance of choosing working alone over cooperating.

## 5. Study 3

Study 3 aimed to investigate the effects of punishment within the game. The CN model predicts that below-threshold stealing will be insensitive to the severity of punishment. CN link this prediction to real-world evidence from criminology. Estimating the elasticity of criminal offending to punishment has proven difficult using observational data, not least because high crime rates cause societies to make their punishments more severe, producing a positive correlation between punishment severity and offending. Studies attempt to identify causal effects by examining the impacts of temporal or spatial variation in sentencing policy, or the transition from juvenile to adult courts (see [14] for a thorough review). Such studies lead to the conclusion that increasing the severity of (already quite large) punishments has at most a modest deterrent effect, and possibly none.

In study 3, we cross-factored the presence or absence of a desperation threshold with the size of the punishment for being caught stealing (lenient or harsh). Both the lenient and harsh punishments were sufficiently large that they rendered the expected payoff from stealing negative. For the threshold condition, CN predicts the harsh punishment to be no more a deterrent than the lenient one. For the no threshold condition, the expected payoff should determine the decision. That is, stealing is clearly a worse decision under the harsh punishment than the lenient one (though, in neither case is stealing generally preferable to not stealing). We thus predicted an interaction between threshold condition and punishment severity, for individuals with below 200 points: in the no-threshold condition, stealing should be rarer under the harsh than the lenient punishment; in the threshold condition, there should be no such difference.

In addition, study 3 allowed us to replicate and further investigate the study 2 interaction between the presence of a threshold and being below the threshold points level. The evidence for that interaction was only anecdotal in study 2. Moreover, we wanted to clarify whether the presence of a threshold reduces stealing above and/or increases it below. We also made other minor procedural changes for study 3 to reduce dropout, optimize incentives and ensure participants understood the rules of the game.

### 5.1. Methods

#### 5.1.1. Experimental game

Studies 3 and 4 incorporated the following modifications to the game. If the participants chose a wrong answer in comprehension questions, instructive feedback was provided as to why the answer was wrong. The number of errors made in the comprehension questions before choosing the right answer was recorded and later used as an exclusion criterion. The number of mock rounds was reduced from 5 to 4, and the random fluctuations of energy points were eliminated. The outcome page from each round displayed the current points level graphically as well as verbally, with the threshold, where applicable, shown as a red line. Each round, in addition to asking how much the player trusted other group members to cooperate, they were also asked how likely other group members were to steal. The two variables were moderately correlated (*r* = 0.44). We use the cooperation trust variable in analysis for comparability with studies 1 and 2. The points and cash values of the different possible situations were adjusted, as shown in table 1.

Rather than starting all participants at the threshold value, in study 3 we drew the initial points levels from the set {180, 190, 200} with equal probability. Having most individuals below threshold this way was to maximize the amount of stealing and hence the power to test hypotheses about punishment. Studies 1 and 2 had already shown the effect of being above or below the threshold on stealing, and hence it was not necessary to power the study optimally to demonstrate that effect.

To maximize power, the two experimental treatments, threshold versus no threshold and harsh versus lenient punishment were implemented within sets. This meant that players encountering one another, unbeknownst to them, were in different conditions. The punishments were −30 and −15 points, respectively.

#### 5.1.2. Data analysis and predictions

The main pre-registered prediction (P3.1) was that increasing punishment severity should have a smaller deterrent effect in the threshold than the no-threshold condition. Several other predictions were made but described as exploratory. These included two replication predictions from studies 1 and 2: an interaction between absence/presence of a threshold and being above/below threshold level in predicting stealing; and trust predicting the choice between cooperating or working alone for those not stealing. The further predictions were exploratory and concerned the fine details of how punishment and stealing work within the game. We present these in a supplementary table (electronic supplementary material, table S1), with just the main and replication predictions reported in the main text.

### 5.2. Results

**Figure 4.**
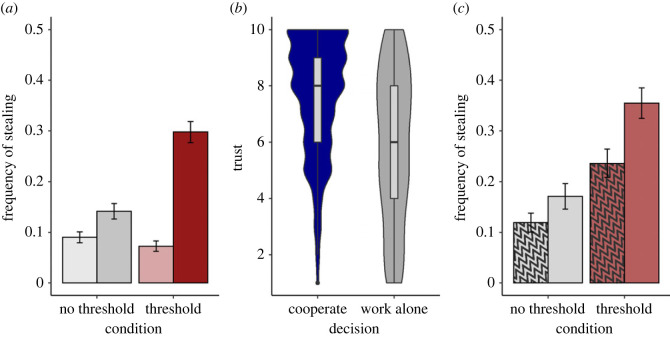
Results of study 3. (*a*) Frequency of stealing, by whether player's energy points level was currently above 200 (light) or below 200 (dark), and condition (threshold versus no threshold). Error bars represent one standard error of the proportion. Analysis collapses across levels of punishment severity. (*b*) Distribution of trust ratings prior to choosing to cooperate, or to work alone. Analysis collapses across levels of punishment severity and threshold condition. (*c*) Probability of stealing for players with fewer than 200 points, by condition and punishment severity (hatched: harsh; open: lenient). Error bars represent one standard error of the proportion.

Collapsing across levels of punishment severity, there was an interaction between the presence/absence of a threshold and whether the points level was above or below 200 in predicting stealing (figure 4*a*; P3.7: OR 3.05, 95% CI 1.38 to 6.75, BF 17.66, strong evidence). Being below 200 points substantially increased stealing in the threshold condition (OR 3.33, 95% CI 1.73 to 6.43, BF 157.53, extreme evidence) but not the no threshold condition (OR 1.01, 95% CI 0.58 to 1.74, BF 0.28, moderate evidence for null). The rate of stealing below 200 points was higher in the threshold than the no threshold condition (OR 2.58, 95% CI 1.23 to 5.36, BF 9.20, moderate evidence). The rate of stealing above 200 points was not substantially lower in the threshold than the no-threshold condition (OR 0.77, 95% CI 0.26 to 2.26, BF 0.60, anecdotal evidence for null). As before, among players not stealing, higher trust increased the likelihood of cooperating rather than working alone (figure 4*b*; OR 3.36, 95% CI 2.56 to 4.46, BF > 1000, extreme evidence).

We predicted (P3.1) an interaction between punishment severity and threshold presence on the likelihood of stealing, for players with fewer than 200 points. That is, increasing punishment severity would be less of a deterrent in the threshold condition. There was no evidence for such an interaction (figure 4*c*; OR 0.58, 95% CI 0.17 to 1.97, BF 0.93, anecdotal evidence for null). However, although harsh punishment tended to reduce stealing overall, the strength of evidence was anecdotal and the credible interval included no effect (OR 0.53, 95% CI 0.26 to 1.10, BF 1.69). Thus, while there was no evidence for the predicted differential sensitivity to punishment severity according to the presence of a threshold, the evidence for *any* sensitivity to punishment severity was weak. Results of further exploratory analyses are presented in electronic supplementary material, table S1.

### 5.3. Discussion

For the replication predictions, critically, we replicated the study 2 finding of an interaction between threshold presence and being below the threshold level, with a stronger level of evidence than in study 2. Moreover, in study 3, the interaction was clearly driven by an *increase* in stealing below the threshold, with no evidence of the threshold causing a decrease in stealing when above it. Thus, study 3 confirmed that the presence of a threshold causes an increase in stealing when the player falls below, but not that the presence of a threshold deters stealing when the player is above it.

The main aim of study 3 was to test the CN prediction that, in the presence of a threshold, below-threshold stealing would be insensitive to the severity of punishment. The trend was in the direction of less stealing where punishment was harsher, and hence contrary to the prediction, although the strength of evidence was anecdotal. Moreover, there was no evidence of an interaction with threshold presence, which we had predicted. Given the substantial sample size and powerful within-set design, we can thus conclude that the sensitivity of below-threshold stealing to punishment severity, across these levels of severity, is at best modest, and does not clearly differ in the presence or absence of a desperation threshold. However, higher-powered experiments with more levels of punishment severity are required to establish the sensitivity to punishment more definitively.

Furthermore, we did not here examine the effect of varying the *probability* of punishment. A long tradition of thought in criminology holds that potential offenders are more sensitive to the probability of punishment than its severity, as long its severity meets a certain threshold of aversiveness [14]. Greater sensitivity to the probability than severity of punishment was also a prediction from the CN model. Moreover, punishments in society can be of many types, not just the taking of material resources. Material punishments are the most easily implementable within an economic game, but sensitivity to punishment severity may vary with punishment type. Therefore, a fuller exploration of punishment in relation to desperation thresholds, including different probabilities and types of punishment, is warranted in future.

## 6. Study 4

Study 4 aimed to test a population-level prediction from CN: greater inequality in resources will lead to more stealing, lower trust, and less cooperation. CN predicts this to be true as long as the greater inequality pushes a larger fraction of the population into a below-threshold position, and as long as there is a desperation threshold. To that end, we manipulated the starting distributions of points. In the equal condition, all participants began with the same points, just above the threshold. In the unequal condition, the mean number of points per participant was the same, but the distribution was dispersed such that half the participants began above the threshold, and half below. We cross-factored the inequality treatment with the presence or absence of a threshold. Study 4 also gave us an opportunity to replicate the interaction between the presence of a threshold and being below the threshold level of points, observed in studies 2 and 3.

### 6.1. Methods

#### 6.1.1. Experimental game

The experimental game was as study 3 (lenient punishment), except for the initial distribution of points. In the equal condition, all players began on 205 points. In the unequal condition, starting levels were drawn evenly from the set {180, 190, 220, 230}. Thus, half the players would begin above the threshold level and half below. Points allocations for the mock and real rounds were independent. Both inequality and threshold presence were varied between sets.

### 6.2. Results

**Figure 5.**
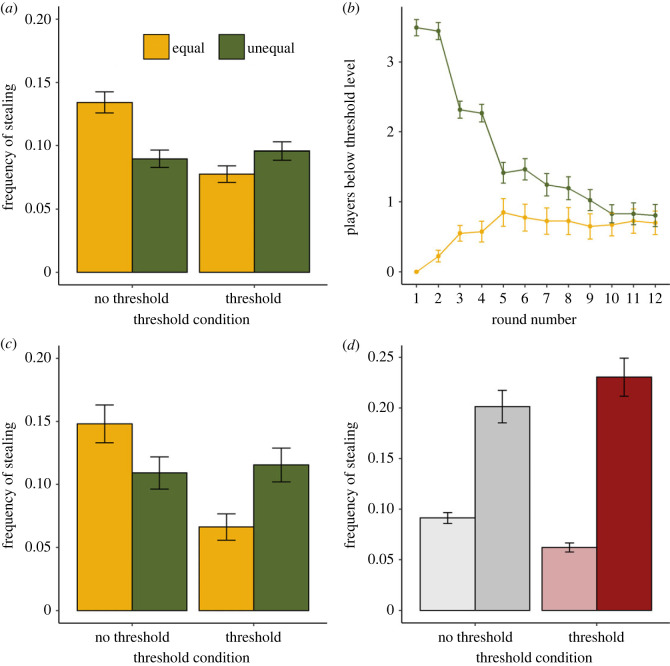
Results of study 4. (*a*) Frequency of stealing by inequality condition and presence of a threshold. Error bars represent one standard error of the proportion. (*b*) Number of players with points below threshold level, by round and inequality condition. Error bars represent one between-set standard error. (*c*) Frequency of stealing by inequality condition and presence of a threshold, first four rounds only. (*d*) Frequency of stealing, by whether player's energy points level was currently above 200 (light) or below 200 (dark), and threshold condition (threshold versus no threshold), in the unequal condition. Error bars represent one standard error of the proportion.

Results for the main pre-registered predictions concerning inequality favoured the null (P4.1–P4.3, P4.5, P4.6) or supported the predictions only anecdotally (P4.4, table 5; figure 5*a*). However, we had failed to anticipate that the difference in inequality between the two conditions would attenuate over the rounds. Specifically, many of the players assigned to begin the game below threshold in the unequal conditions managed to regain the threshold over time, while some players in the equal condition fell below. After round four, the difference between conditions in the number of players below threshold level was very small (figure 5*b*). Four rounds is indeed the number required for initially below-threshold players to regain the threshold through cooperation. We therefore repeated the planned analyses but on the data for the first four rounds only.

**Table 5.** Pre-registered predictions and corresponding results, study 4, for all rounds, and the first four rounds only.

number	prediction	result (all rounds)	result (first four rounds)
P4.1	in the presence of a threshold, sets in the unequal condition will have higher rates of stealing than those in the equal condition	anecdotal evidence for null. OR 1.53 (95% CI 0.67–3.52), BF 0.71	anecdotal supporting evidence. OR 1.96 (95% CI 0.98–3.98), BF 2.23
P4.2	in the presence of a threshold, sets in the unequal condition will develop lower trust than equal sets, not confined to individuals whose resources are below the threshold	moderate evidence for null. *β* = 0.01 (95% CI −0.29 to 0.32), BF 0.15	moderate evidence for null. *β* = 0.03 (95% CI −0.25 to 0.30), BF 0.14
P4.3	in the presence of a threshold, sets in the unequal condition will have lower rates of cooperation than those in the equal condition	anecdotal evidence for null. OR 0.87 (95% CI 0.31 to 2.42), BF 0.54	anecdotal evidence for null. OR 0.80 (95% CI 0.32 to 1.99), BF 0.51
P4.4	the difference in rate of stealing between equality and inequality conditions will be larger when there is a desperation threshold than when there is no threshold	anecdotal supporting evidence. OR (interaction) = 2.16, 95% CI 0.72 to 6.40, BF 1.47	moderate supporting evidence. OR (interaction) = 2.57, 95% CI 0.87 to 7.43, BF 2.50
P4.5	the difference in trust between equality and inequality conditions will be larger when there is a desperation threshold than when there is no threshold	moderate evidence for null. *β* (interaction) = −0.15 (95% CI −0.55 to 0.25), BF 0.26	moderate evidence for null. *β* (interaction) = −0.11 (95% CI −0.48 to 0.27), BF 0.22
P4.6	the difference in rate of cooperation between equality and inequality conditions will be larger when there is a desperation threshold than when there is no threshold	anecdotal evidence for null. OR (interaction) = 0.56 (95% CI 0.17 to 1.87), BF 0.97	anecdotal supporting evidence. OR (interaction) = 0.51 (95% CI 0.16 to 1.62), BF 1.15

Considering the threshold condition alone, there was more stealing in the first four rounds in the unequal than equal condition, with an odds ratio of almost two (figure 5*c*; table 5). However, trust was not lower, and nor was cooperation less frequent. Considering both threshold and no threshold conditions, in the first four rounds there was moderate evidence for an interaction between threshold presence and inequality condition on stealing. In the threshold condition, as mentioned above, there was more stealing in the unequal than equal condition. In the no-threshold condition, the evidence anecdotally favoured the null of no difference (OR = 0.63, 95% CI 0.23 to 1.75, BF 0.77; figure 5*c*).

Comparing to study 3, we found a similar interaction between being below 200 points and the presence of a threshold on stealing, albeit with a smaller effect size (OR (interaction) = 1.82, 85% CI 1.00–3.28, BF 2.16, anecdotal evidence; figure 5*d*; this analysis includes all rounds since it does not concern the inequality treatment). Being below 200 points increased stealing more in the presence of a threshold (OR 2.75, 95% CI 1.71 to 4.37, BF 963.46 extreme evidence) than it did in the absence of one (OR 1.32, 95% CI 0.88 to 1.98, BF 0.52, anecdotal evidence for null). The rate of stealing above 200 points was not clearly different in the threshold and no-threshold conditions (OR 0.62, 95% CI 0.31 to 1.22, BF 0.91, anecdotal evidence for null).

### 6.3. Discussion

In study 4, we tested the CN prediction that greater inequality in resources, by causing some players to be below the threshold level, would produce more stealing, lower cooperation, and less trust. CN predicts this only in the presence of a threshold. In the pre-registered analysis of all the data, these predictions were not clearly supported. However, the reasons for this failure are easily interpretable: our experimental treatment did not in fact create greater inequality for the full course of the experiment. We manipulated only the initial distribution of resources; as players reaped the payoffs of their actions over rounds, the two conditions converged. When we used the data from just the first four rounds—i.e. the part of the experiment during which the ‘unequal’ condition was more unequal in a consequential way—there was, as predicted, more stealing in unequal than equal sets, only in the threshold condition. Thus, this analysis, although departing from our pre-registration, supports the hypothesis that an increase in inequality that puts some people below threshold level, when coupled with a desperation threshold, produces more theft. However, our hypotheses concerning trust and cooperation were not supported even in the first four rounds: despite there being more stealing in the unequal groups in the early rounds, trust was not lower and cooperation was no less frequent. It is possible that the inequality difference between conditions was too transient for these effects, which we expected given the relationships between theft, trust and cooperation observed in studies 1–3, to be seen.

Study 4 also gave us an opportunity to replicate the interaction between having fewer than 200 points and the presence of a threshold seen in study 3. As there, being below 200 points sharply increased stealing, but only if 200 points was a desperation threshold, and not otherwise.

## 7. General discussion

We developed an experimental framework in which to test key predictions of the CN model of desperation and stealing. Those key predictions were four: (i) having a level of resources that puts one below a desperation threshold increases stealing from cooperators; (ii) more desperation in a population, by producing more stealing, reduces trust and makes people avoid interaction; (iii) below-threshold stealing is insensitive to the magnitude of punishment for being caught; and (iv) greater resource inequality in a population, because it increases the fraction of individuals with resource levels below the threshold, produces more stealing, lower trust and lower cooperation. Within the experimental context we created, predictions (i), (ii) and (iv) were supported (though see below for nuance), whereas the evidence tended against (iii).

For prediction (i), in all four experiments, individuals were more likely to steal when their resource levels fell below threshold. In experiments 2–4, by including a no-threshold condition, we were able to show that this increased stealing was not merely due to having lost ground relative to a starting or reference point. The effect of having fewer than 100 points (study 2) or 200 points (studies 3 and 4) on stealing was clear in the threshold conditions, but weak in the no threshold conditions. Below-threshold participants are in a predicament that will cost them dramatically if they do not rectify the situation as soon as possible. As in previous experimental work [15], this induced the taking of risks: here, the risk inherent in stealing from another player under the possibility of punishment.

The evidence for prediction (ii) was consistent across all studies, supporting the idea that trust is an internal regulatory variable tracking the propensity of others in the community to cooperate, and hence the propitiousness of social engagement, in the local time and place [25]. Importantly, the lower trust in sets containing people with below-threshold resource levels affected the whole set, not just the below-threshold individuals themselves.

Prediction (iii), insensitivity to punishment, is where our experimental results most clearly differ from the predictions of CN. We used two levels of punishment, and the harsher one tended to produce less stealing than the lenient one for individuals below the desperation threshold. The evidence for sensitivity was only anecdotal by standard BF thresholds [24]. Nonetheless, this result is at odds with CN's predictions. Further investigation of prediction (iii), including a greater variety of magnitudes as well as probabilities of punishment, is warranted. For example, in the present experiment, the harsh punishment was actually greater than the penalty for being below the threshold for a round. It is possible that people would be insensitive to variation in punishment severity in the range of punishments that are smaller than the desperation penalty.

For prediction (iv), the effects of inequality, the results integrated across the four experiments were generally supportive, with caveats regarding study 4 (see below). CN's causal argument is as follows: (a) increasing inequality increases the fraction of individuals with below-threshold resource levels; (b) more below-threshold resource levels produces more stealing; (c) more stealing leads to low trust on the part of other players; (d) lower trust leads to withdrawal from cooperation. All these causal linkages are present in our experimental results. For example, in experiments 1 and 2, a larger fraction of individuals with below-threshold resource levels was robustly associated with more stealing (linkage b) and lower trust (linkage c); and in all experiments, lower trust predicted more working alone and less cooperation (linkage d). Study 4 was the most direct test of prediction (iv). Although there was some support for the prediction, the caveats are as follows. First, the pre-registered analysis of all the rounds supported the null for the effect of inequality on stealing in the presence of a threshold (linkage b). In exploratory analysis, we found that the number of below-threshold players in the ‘unequal’ sets had only been markedly greater for the first few rounds, after which the two conditions converged. The fact that we found more stealing in the inequality condition *only* in those early rounds if anything strengthens the inference that inequality causes more stealing. The second caveat on study 4 is that we did not see effects of inequality on trust and cooperation, either in the first four or in all the rounds. We expected these effects given that earlier studies had shown that more stealing lowers trust and hence reduces cooperation. Because the condition difference in number of players below threshold was transitory, the effect of inequality on stealing was small, which may explain why it did not feed through to trust or cooperation.

A key limitation of our experimental approach, given the real-world phenomena we seek to model, is that the stakes are low and the game brief. The worst jeopardy we can place our participants into is the loss of a small cash bonus that may not even have been their prime motivation for taking part, and makes at most modest difference to their state. Clearly, this restricts the informativeness of our results with regard to real material deprivation. While fully acknowledging this limitation, we place a more positive light on it: if even in a brief, low-stakes set of anonymous voluntary interactions we can show that being below a resource desperation threshold induces stealing, and that stealing reduces trust and destroys cooperation, then the plausibility of this being an important causal sequence in real societies, where stakes are much higher and the game persists, is enhanced. Likewise, our relatively high-SES, educated participant pool is not representative of people who find themselves in genuine material desperation, or indeed commit acquisitive crimes. Again, we would place a positive interpretation on this limitation: the results show that any of us could be tempted to steal when faced with certain material contingencies. Nonetheless, future research could apply the present paradigm to a broader range of participants, and measure societal, sociodemographic and individual-difference variables that might moderate the response to the experimental situation.

Our results are consistent with the idea that the link between low material resources and criminal offending is causal, a contention also supported, for example, by the results of cash transfer policies on offending [27]. More specifically, we found support for our earlier theoretical claims that the existence of a desperation threshold could exacerbate the effect of material scarcity on acquisitive crime. A key outstanding question is therefore whether desperation thresholds exist in the lived experience of people facing adversity. It is extremely well established that there are diminishing marginal returns to income and wealth [28–30]. This implies a steep wellbeing gain from additional resources at a certain point. The desperation threshold as modelled in CN and implemented here also includes a zone where there is effectively ‘nothing left to lose’ (i.e. where the cash earnings will hit zero unless the person does something dramatic, and cannot get any worse than zero). It is a priority to investigate whether, in reality, the functions linking people's social wellbeing to their resources have this dual shape, or at least, whether people perceive that they do. If the assumption that there are resource desperation thresholds in people's real-world experience is shown to be an adequate one, then the current experiments demonstrate some of the behavioural consequences that are likely to follow, and the implications of these for society.

## Data Availability

Data and analysis code are available at: https://osf.io/kf87e/. The data are provided in electronic supplementary material [31].
